# Genetic and physiological basis for antibody production by *Kluyveromyces marxianus*

**DOI:** 10.1186/s13568-018-0588-1

**Published:** 2018-04-12

**Authors:** Yumiko Nambu-Nishida, Keiji Nishida, Tomohisa Hasunuma, Akihiko Kondo

**Affiliations:** 1 Technology Research Association of Highly Efficient Gene Design (TRAHED), 7-1-49 Minatojimaminamimachi, Chuo-ku, Kobe, Hyogo 650-0047 Japan; 2 Department of Chemical Science and Engineering, Graduate School of Engineering, Kobe University, 1-1 Rokkodai-cho, Nada-ku, Kobe, Hyogo 657-8501 Japan; 3 Graduate School of Science, Technology and Innovation, Kobe University, 1-1 Rokkodai-cho, Nada-ku, Kobe, Hyogo 657-8501 Japan

**Keywords:** *Kluyveromyces marxianus*, Single-chain antibody (scFv), *INU1*, Inulinase, MgSO_4_

## Abstract

*Kluyveromyces marxianus* is a thermotolerant, crabtree-negative yeast, which preferentially directs metabolism (e.g., from the tricarboxylic acid cycle) to aerobic alcoholic fermentation. Thus *K. marxianus* has great potential for engineering to produce various materials under aerobic cultivation conditions. In this study, we engineered *K. marxianus* to produce and secrete a single-chain antibody (scFv), a product that is highly valuable but has historically proven difficult to generate at large scale. scFv production was obtained with strains carrying either plasmid-borne or genomically integrated constructs using various combinations of promoters (P_*MDH1*_ or P_*ACO1*_) and secretion signal peptides (KmINUss or Scα-MFss). As the wild-type *K. marxianus* secretes endogenous inulinase predominantly, the corresponding *INU1* gene was disrupted using a Clustered Regularly Interspaced Short Palindromic Repeat (CRISPR)—associated protein (CRISPR–Cas9) system to re-direct resources to scFv production. Genomic integration was used to replace *INU1* with sequences encoding a fusion of the INU1 signal peptide to scFv; the resulting construct yielded the highest scFv production among the strains tested. Optimization of growth conditions revealed that scFv production by this strain was enhanced by incubation at 30 °C in xylose medium containing 200 mM MgSO_4_. These results together demonstrate that *K. marxianus* has the potential to serve as a host strain for antibody production.

**Electronic supplementary material:**

The online version of this article (10.1186/s13568-018-0588-1) contains supplementary material, which is available to authorized users.

## Introduction

Production of biopharmaceuticals requires the difficult choice of a host cell capable of generating the desired product in an active and safe form, devoid of unwanted modification or contamination. Additionally, some biopharmaceuticals such as antibodies have proven difficult to express at high levels. Chinese hamster ovary (CHO) cells and yeasts are the major hosts that have been engineered to produce biopharmaceutical products, including antibodies (Maccani et al. 2014). As mammalian cells, CHO cells produce mammalian-derived proteins in an active form, bearing appropriate modifications such as glycosylation. However, the development of stable cell lines takes very long times (6–12 months), and the cost of cell culture is very high (Lai et al. 2013). Bacterial expression host such as *Escherichia coli* provides much cheaper option, while proteins that require eukaryotic post-translational modifications are not suitable (Swartz 2001; Jevševar et al. 2005). Yeast cells such as *Pichia pastoris* may provide much faster and cheaper ways of production (Çelik and Çalık 2012); while this yeast can be engineered to serve as a suitable hosts, highly complex proteins such as antibodies can be difficult to express efficiently in this system (Nielsen 2013). In the previous study, we have shown that *Kluyveromyces marxianus* grow faster than *Saccharomyces cerevisiae* at wider range of temperature (Nambu-Nishida et al. 2017). *K. marxianus* also does not show obligate ethanol production aerobically and thus is expected to be engineered to produce various substrates (Wagner and Alper 2016).

Due to difficulties in expression, secretion, and post-translational modification, antibodies intended for clinical use remain a challenge to produce in a cost-effective manner (Buckholz and Gleeson 1991; Huang et al. 2014). Single-chain Fv antibody (scFv) is one of the most useful forms of antibody, consisting of a single polypeptide in which the variable regions of the heavy (V_H_) and light (V_L_) chain domains are fused by a short, flexible linker; the resulting product has a molecular weight of approximately 30 kDa (Damasceno et al. 2004). Unlike large immunoglobulins (IgGs), scFv proteins have demonstrated rapid tumor penetration (Yokota et al. 1992). A prototypical scFv is the anti-chicken (anti-hen) egg white lysozyme antibody (HyHEL-10), which has been used for the precise analysis of antigen–antibody interactions (Tsumoto et al. 1997).

The non-conventional yeast *Kluyveromyces marxianus* can grow on various sugars (glucose, xylose, fructose, sucrose, inulin, etc.) (Fonseca et al. 2008; Lane and Morrissey 2010; Lertwattanasakul et al. 2011). *K. marxianus* is known to secrete proteins such as inulinase into the culture medium at high levels (Rouwenhorst et al. 1990; Hu et al. 2012). Engineering of *K. marxianus* for protein production has been reported for both endogenous and heterologous enzymes (Raimondi et al. 2013; Hong et al. 2007). However, there are to date (to our knowledge) no reports on secretory antibody production in *K. marxianus*.

The *K. marxianus* NBRC1777 strain recently has been shown to exhibit rapid growth and adaptability to a wide range of temperatures (from 5 to 45 °C). Additionally, comprehensive genome engineering tools recently have been introduced for use in this strain, including a Clustered Regularly Interspaced Short Palindromic Repeat (CRISPR)—associated protein (CRISPR–Cas9) system and deaminase-mediated base editing Target-AID (Nambu-Nishida et al. 2017). NBRC1777 is expected to be of use for various bio-production applications, including the secretion of high-value proteins.

In the present study, we introduced *K. marxianus* NBRC1777 as a novel host for scFv production. Several parameters were examined, including the type of secretion signal and growth conditions such as temperature, carbon source, and medium. Genetic backgrounds that affect protein production or secretion also were studied.

## Materials and methods

### Strains and culture conditions

The *K. marxianus* and *S. cerevisiae* strains used in this study are listed in Table 1. *E. coli* strain DH5α (Toyobo, Osaka, Japan) was used for vector construction and cloning. *E. coli* and yeast cells were grown as described previously (Nambu-Nishida et al. 2017). Genomic DNA from *S. cerevisiae* BY4741 was used as a template to amplify α-MF (Scα-MF) coding fragment.

**Table 1:** Plasmids and strains used in this study

Plasmids and strains	Genotype	References
Plasmids		
E02-012	KmP_*MDH1*__KmINU1ss_scFv_T_*TDH3*_, KmARS7, KmCEN D, kanMX, ori, and AmpR	This study
E02-014	KmP_*ACO1*__KmINU1ss_scFv_T_*TDH3*_, KmARS7, KmCEN D, kanMX, ori, and AmpR	This study
E02-020	KmP_*MDH1*__Scα-MFss_scFv_T_*TDH3*_, KmARS7, KmCEN D, kanMX, ori, and AmpR	This study
E02-022	KmP_*ACO1*__Scα-MFss_scFv_T_*TDH3*_, KmARS7, KmCEN D, kanMX, ori, and AmpR	This study
Cas9_Base	ScP_*PDC1*__Cas9_T_*TDH3*_, KmARS7, KmCEN D, *kanMX*, ori, and AmpR	Nambu-Nishida et al. (2017)
E02-025	KmP_*SNR52*__target_gRNA-1_sgRNA_T_*sup4*_ cassette and KmP_*SNR52*__target_gRNA-3_sgRNA_T_*sup4*_ cassette in Cas9_Base	This study
E02-026	KmP_*SNR52*__target_gRNA-1_sgRNA_T_*sup4*_ cassette and KmP_*SNR52*__target_gRNA-2_sgRNA_T_*sup4*_ cassette in Cas9_Base	This study
Strains		
1 (NBRC1777)	Wild-type (WT)	NITE Biological Resource Center, Japan
*Nej1°*	*Nej1* disrupted by C to T point mutation at posision 13	Nambu-Nishida et al. (2017)
*Dnl4°*	*Dnl4* disrupted by G to A point mutation at posision 44	Nambu-Nishida et al. (2017)
2 (Km02-026)	WT/E02-012	This study
3 (Km02-032)	WT/E02-014	This study
4 (Km02-050)	WT/E02-020	This study
5 (Km02-056)	WT/E02-022	This study
Δ*inu1* (Km02-063)	*Nej1°*/Δ*inu1*	This study
6 (Km02-064)	*Nej1°*/*inu1::*P_*INU1*__scFv	This study
7 (Km02-065)	*Dnl4°*/*inu1::*P_*INU1*__scFv	This study
8 (Km02-066)	*Nej1°*/*inu1::*/E02-020	This study
BY4741	*MATa his3*Δ*1 leu2*Δ*0 met15*Δ*0 ura3*Δ*0*	ATCC (Brachmann et al. 1998)

### *INU1* gene disruption

**Fig. 1:**
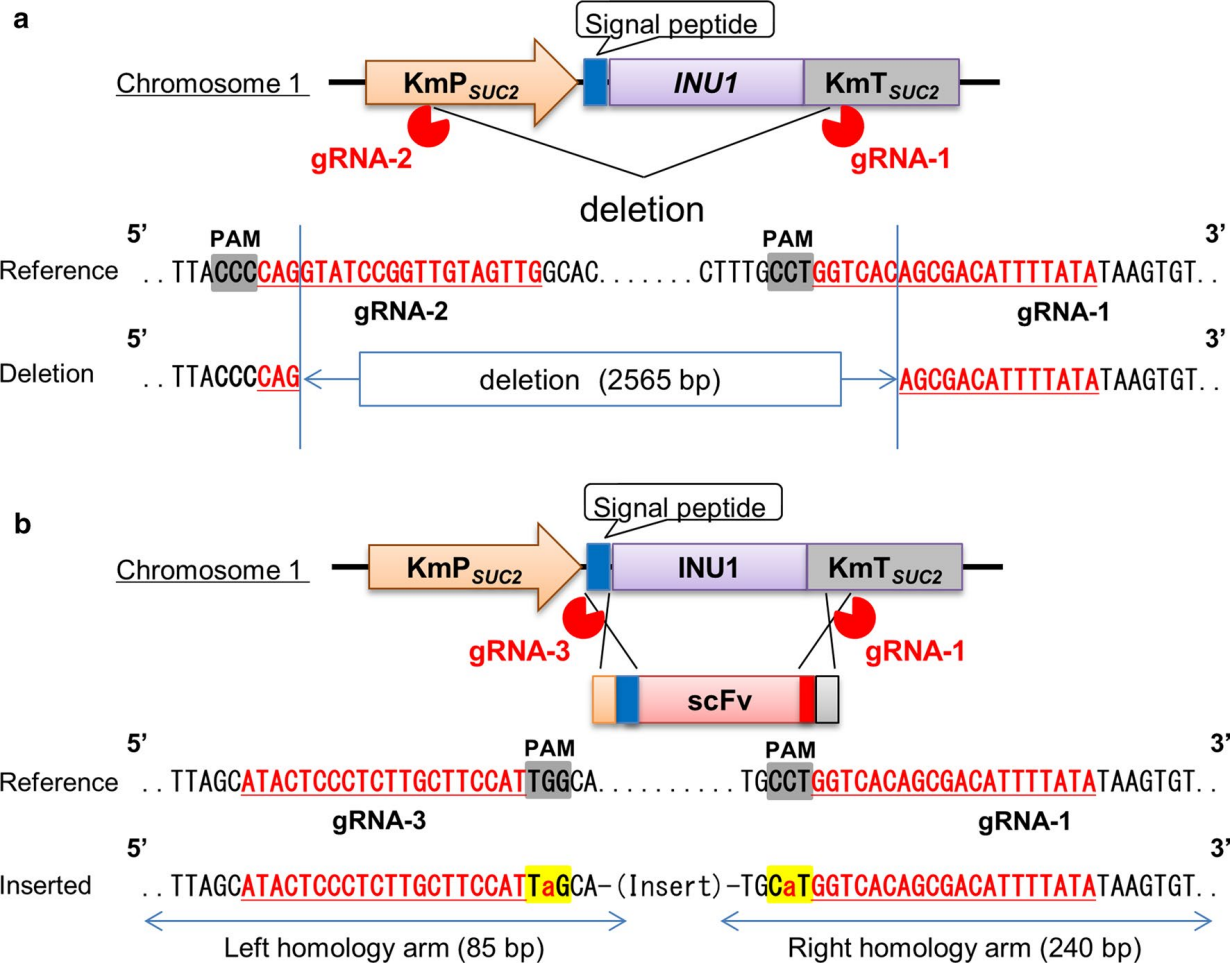
Design of *INU1* disruption and replacement. **a** A schematic of the *INU1* locus and its deletion is shown. Reverse complement sequences of target (red) and PAM (shadowed) are shown on the reference sequence. The lower part of the panel shows an alignment of the sequence from the deletion strain with the sequence of the wild-type locus. **b** A schematic of the *INU1* replacement by a fragment encoding scFv is shown. The mutated PAM sequence and its reverse complement sequence are highlighted in yellow. Mutated bases are indicated as small red letters. The scFv-encoding fragment also encodes the INU1 secretion signal peptide (blue box) and a 6-histidine tag (red box) in-frame with the expressed ORF

The *inu1* gene-disrupted strain and homologous recombination strains were generated using the CRISPR–Cas9 system. The CRISPR–Cas9 vector plasmid (Cas9_Base) of *K. marxianus*, target sgRNA cassette construction, and methods were as described previously (Nambu-Nishida et al. 2017). The *inu1* deletion strain was generated by using a Cas9 plasmid (E02-026) containing gRNA-1 and gRNA-2 target sequences (Table 2 and Fig. 1a).

**Table 2:** Target sequences used in this study

Name	Sequence (5′ → 3′)
gRNA-1	TATAAAATGTCGCTGTGACC
gRNA-2	CAACTACAACCGGATACCTG
gRNA-3	ATGGAAGCAAGAGGGAGTAT

### Replacement of *INU1* by integration of a scFv-encoding sequence

HyHEL-10 scFv (scFv hereafter) amino acid sequence (Additional file 1: Figure S1) was codon optimized for expression in *K. marxianus*. The homologous recombination strain was generated by transforming the recipient strain by the lithium acetate method (Gietz et al. 1992), using 10 µg of Cas9 plasmid (E02-025) containing gRNA-1 and gRNA-3 target sequences (Table 2 and Fig. 1b) and 5 µg of the sequence-optimized fragment encoding scFv (Additional file 1: Figure S2). The transformed cells were plated on YPD containing the appropriate selection reagent (100 μg/mL G418).

### Verification of genome-edited cells

Transformants generated using the CRISPR–Cas9 system were screened by colony PCR using the primer pair P_Km01-010 + P_Km01-010-011 (Additional file 1: Table S1). DNA sequence of the resulting amplicon was confirmed by sequencing using a 3130xL Genetic Analyzer (Applied Biosystems, CA, USA). The transformant cells were grown without selection reagents to isolate a clone that dropped the Cas9 plasmid.

### Construction of scFv expression plasmids

The constructed plasmids are listed in Table 1. Plasmid E02-014, which includes KmARS7, KmCEN-D, the scFv-encoding fragment, KmP_*ACO1*_, and the *kanMX* selectable marker (which provides G418 resistance) is shown as an example (Additional file 1: Figure S3).

Constructs incorporated either the P_*MDH1*_ (KmP_*MDH1*_) (Additional file 1: Figure S4) or P_*ACO1*_ (KmP_*ACO1*_) (Additional file 1: Figure S5) promoters from *K. marxianus*. Constructs also incorporated sequences encoding either the secretory signal sequence from inulinase (KmINUss) from *K. marxianus* (Bergkamp et al. 1993) or that from Scα-MFss from *S. cerevisiae* (Melorose et al. 1986). Fragments carrying the desired promoter fragment and encoding the desired signal sequence were inserted into the *Nhe*I or *Sbf*I/*Bam*HI sites of the E02-014 plasmid using In-fusion cloning (Takara Bio, Shiga, Japan). The resulting scFv expression plasmids were transformed into *K. marxianus* NBRC1777 or the *inu1* deletion strain by the transformation and selection methods noted above.

### SDS-PAGE and immunoblot analysis

To analyze protein production, soluble proteins in the spent culture medium were separated on a SDS-polyacrylamide 12.5% gel (ATTO, Tokyo, Japan) and stained with Bio-Safe Coomassie Stain (Bio-Rad, Hercules, CA). MagicMark™ XP Western Protein Standard (Thermo Fisher Scientific Inc., MA, USA) was included as the molecular weight standard. For western blotting, proteins were transferred to a polyvinylidene fluoride (PVDF) membrane (EDM Millipore, Billerica, MA, USA) by electroblotting. The membrane then was blocked by incubation for 1 h at room temperature with Blocking One (Nacalai tesque, Kyoto, Japan), followed by washing with TBST (0.1 M Tris–HCl, 0.15 M NaCl, 0.05% Tween 20). The membrane then was incubated for 1 h with the primary antibody, rabbit anti-6-His Antibody Affinity Purified (Bethyl Laboratories, TX, USA) diluted 1:5000, followed by washing with TBST and incubation for 1 h with the secondary antibody, Peroxidase AffiniPure Goat Anti-Rabbit IgG (H+L) (Jackson ImmunoResearch Laboratories, PA, USA) diluted 1:10,000. Protein bands were detected by ImmunoStar Zeta (Wako, Osaka, Japan).

### Enzyme-linked immunosorbent assay (ELISA)

Strains were cultured in YPD or YPX (10 g/L yeast extract, 20 g/L peptone, and 20 g/L xylose) supplemented with 100 mM sodium phosphate buffer, pH 6.0, and selective agent for plasmid-bearing strains in the absence or presence of 200 mM MgSO_4_ selective medium. Culturing was performed in 96-well deep-well plates at 20 or 30 °C with shaking at 1200 rpm.

For ELISA, a MaxiSorp plate (Thermo Fisher Scientific Inc.) was coated by distribution of 50 μL per well of lysozyme formulated at 1 μM in 1× phosphate-buffered saline [PBS (10× stock), Nacalai Tesque] followed by overnight incubation at 4 °C. The plate then was blocked at 25 °C for 1 h with the blocking solution (ImmunoBlock, DS Pharma Biomedical, Japan) diluted 1:5 in water. The plate was washed three times with PBST (1× PBS supplemented with 0.1% Tween-20 and 2% blocking solution). Supernatants (spent medium) from cultures were diluted fivefold in PBST containing 2% ImmunoBlock and distributed at 50 μL/well. All ELISAs included a blank consisting of 70 μL PBST containing 2% ImmunoBlock. Following incubation at 25 °C for 1 h, the plate was washed as above, and antibody (Anti-His-tag mAb-HRP-DirecT, MLB, Nagoya, Japan), diluted 1:8000 in PBST, was distributed at 50 μL/well. The plate was incubated at 25 °C for 1 h and then washed with PBST as above. Color was developed using TMB 1-Component Microwell Peroxidase Substrate Sure Blue and TMB Stop Solution (KLP Inc, Milford, USA) according to manufacturer’s instructions. Activity and growth were then measured as absorbance at 450 nm (ABS_450_) and 600 nm (OD_600_), respectively using a SpectraMax Paradigm Multi-Mode Microplate Reader (Molecular Devices Japan, Tokyo, Japan). Relative activity of scFv was obtained by subtracting the value of blank.

### Accession numbers

The codon-optimized scFv-encoding sequence was submitted to the DDBJ/EMBL/GenBank databases under accession number LC369677. The genome sequence of *K. marxianus* NBRC1777 was in the DDBJ/EMBL/GenBank databases under accession number AP014599 to AP014607 (Inokuma et al. 2015).

## Results

### Disruption of *INU1* gene by CRISPR–Cas9 system

**Fig. 2:**
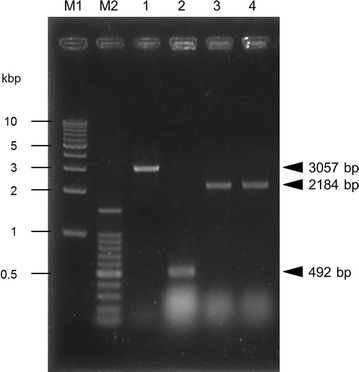
*INU1* deletion and replacement analyzed by agarose gel electrophoresis. Sequences from the transformed cells were PCR-amplified and analyzed by agarose gel electrophoresis. A pair of primers (P_Km01-010 + P_Km01-011) flanking the *INU1* locus were used to amplify intervening sequences. The resulting amplicons from the wild-type strain (lane 1), *inu1*-deleted strain Δ*inu1* (Km02-063) (lane 2), or scFv integration strains No. 6 (Km02-064) (lane 3) and No. 7 (Km02-065) (lane 4) matched the expected fragment sizes of 3057, 492, 2184, and 2184 bp, respectively. DNA ladder markers (1 Kb and 100 bp) are provided as size standards (lanes M1 and M2, respectively)

**Fig. 3:**
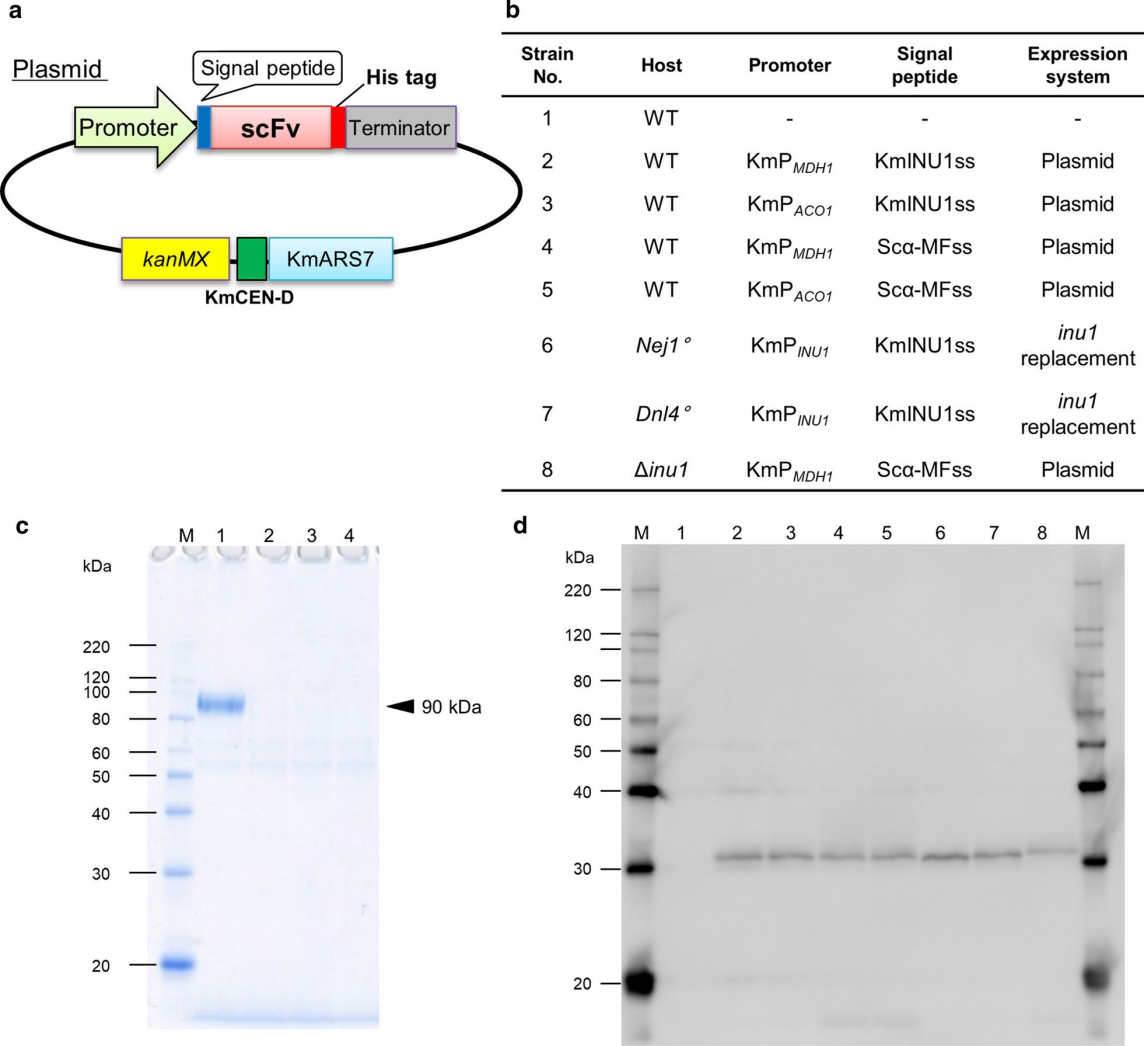
*INU1* deletion analyzed by SDS-PAGE and secretion of scFv analyzed by immunoblotting. **a** Schematic of the general construction of scFv-expression plasmid. *kanMX*: G418 resistance gene, KmCEN-D: centromere sequence of *K. marxianus*, KmARS7: autonomous replication sequence of *K. marxianus*. **b** Combinations of promoters and signal peptides tested. **c** Stains were cultivated for 48 h in YPD with or without G418, and SDS-PAGE was used to analyze the spent culture medium from strains No. 1 (wild-type) (lane 1), No. 6 (Km02-064) (lane 2), No. 7 (Km02-065) (lane 3), and No. 8 (Km02-066) (lane 4). Sizes of the molecular weight marker (lane M) are indicated on the left. **d** Immunoblot of spent culture medium from strains cultivated for 72 h in YPD with or without G418. Lane numbers correspond to those in panel (b)

Wild-type *K. marxianus* predominantly secretes inulinase (Rouwenhorst et al. 1988; Hu et al. 2012). To facilitate the purification of the heterologous protein and re-direct cellular resources for protein production, we deleted the corresponding *INU1* gene. A CRISPR–Cas9 vector for *K. marxianus* (Nambu-Nishida et al. 2017) expressing a pair of guide RNAs (gRNA-1 and gRNA-2) flanking the *INU1* coding region was constructed and used to transform the parent strain; transformants were then screened for the *inu1* mutation (Fig. 1a). For integration of the scFv-encoding sequence at the *INU1* locus, we employed strains deficient in the non-homologous end-joining (NHEJ) repair pathway (in this instance, harboring *nej1°* or *dnl4°* null mutations) (Nambu-Nishida et al. 2017) to facilitate homology-directed integration. Another vector expressing a pair of guide RNAs (gRNA-1 and gRNA-3) flanking the *INU1* coding region (Fig. 1b) was designed and transformed in combination with an scFv-encoding fragment. The scFv-encoding fragment was flanked with arms (85 and 240 bp for the upstream and downstream sequences, respectively) with homology to the *INU1* ORF. PAM sequences of the targets in the arms were mutated to prevent re-cutting after successful integration. Vector-carrying transformant cells were PCR-amplified and subjected to agarose gel electrophoresis (Fig. 2). Sequence analysis of the deletion transformant confirmed that the chromosomal *INU1* locus harbored a 2565-bp deletion between the gRNA-2 and gRNA-1 targeting sites, yielding Δ*inu1* (Km02-063) strain (Fig. 1a). Sequence analysis of the transformants from the gene-replacement experiment confirmed that the *INU1* ORF had been replaced by sequences encoding scFv; two of the resulting constructs were designated strains No. 6 (Km02-064) and No.7 (Km02-065) (Figs. 1b and 3b). Note that these two strains include *nej1°* or *dnl4°*, respectively. Next, the strains were assessed for inulinase secretion.

SDS-PAGE analysis of spent culture medium recovered from the wild-type strain revealed a single major band at approximately 90 kDa (Fig. 3c), consistent with the expected size of inulinase (Hong et al. 2014). Notably, this band was absent in spent medium from cultures of the *inu1* constructs, as expected (Fig. 3c).

### Expression and secretion of scFv antibody

For the expression of scFv, various expression cassettes containing combinations of promoters (P_*MDH1*_, P_*ACO1*_) and secretion signal (KmINUss, Scα-MFss) -sequences were introduced, either via plasmid or by genomic integration (Fig. 3a, b). Protein expression and secretion was assessed by immunoblotting of spent growth medium (Fig. 3d). Secretion of scFv was confirmed as the presence of an approximately 30 kDa protein (Damasceno et al. 2004) in the spent growth medium from each of the transformed strains tested (Fig. 3d). These results indicated that these promoters and secretion signals functioned in a modular fashion.

### Activity of scFv antibody and improved production by magnesium sulfate supplementation

**Fig. 4:**
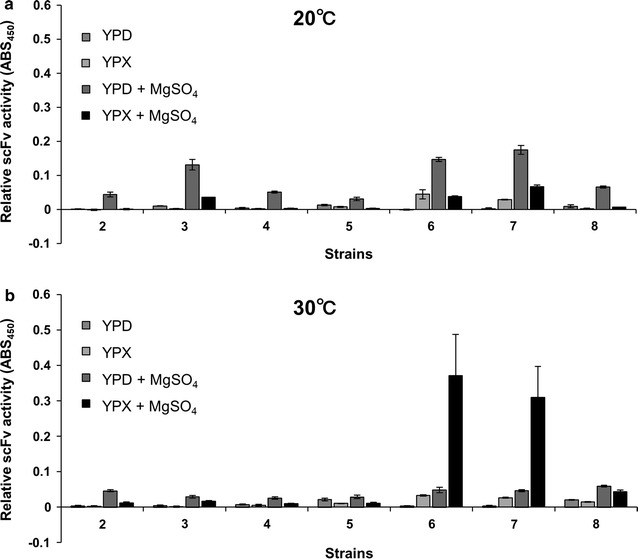
Immunoreactivity of secreted scFv analyzed by ELISA. Strains were cultured in YPD or YPX in the absence or presence of MgSO_4_ at 20 °C (**a**) or 30 °C (**b**). Spent culture medium was harvested at 72 h and subjected to ELISA using lysozyme as an antigen for scFv. Values are presented as mean ± SEM from three independent experiments

We next sought to identify growth conditions, including the use of various media, that would yield enhanced expression and secretion of the intact scFv protein. Cells were grown, with shaking in 96-well deep-well plates, at temperatures of 20 or 30 °C in YPD or YPX medium in the presence or absence of various supplements and subjected to ELISA to detect the presence of intact secreted scFv. In this context, ELISA measured the immunoreactivity of scFv. MgSO_4_ was found to have substantial impact on the antibody production in *K. marxianus* (Fig. 4a, b). At 20 °C, all strains exhibited increased activity when grown in YPD plus MgSO_4_ (Fig. 4a). At 30 °C, more than tenfold increased activity was observed for strains No. 6 (Km02-064) and No. 7 (Km02-065), both of which harbor constructs introduced by genomic integration, when grown in xylose medium containing MgSO_4_ (Figs. 3b and 4b). Deletion of *INU1* yielded about 4.4-fold increase in scFv activity in xylose medium containing MgSO_4_, when comparing strain No. 4 (Km02-050) (harboring a plasmid-borne construct) and strain No. 8 (Km02-066) (*INU1* disruptant harboring a plasmid-borne construction) (Figs. 3b and 4b). The scFv activity (ABS_450_) per cell amount (OD_600_) was calculated and shown in Additional file 1: Figure S6. At 30 °C, the strains No. 6 and No. 7 showed the highest scFv activity per cell amount in YPD plus MgSO_4_ (Additional file 1: Figure S6b).

## Discussion

In this study, we demonstrated that *K. marxianus* NBRC1777 can be engineered to express and secrete a single-chain antibody. We showed that secretion of scFv could be changed substantially by use of various genetic constructs and by modification of the growth conditions.

The recently introduced CRISPR–Cas9 and Target-AID genome editing systems (Nambu-Nishida et al. 2017) permit genetic manipulation of organisms that had previously been underexploited because of a lack of genetic tools. In the present work, a sequence encoding scFv was integrated into the *INU1* locus without an associated selection marker. This construct allowed robust expression of the integrated gene without a need for continued use of selection reagents. The strains (Nos. 6 and 7) carrying the integrated construct showed dramatic increases in scFv immunoreactivity compared to strains expressing scFv via plasmid-borne constructs when grown in YPX plus MgSO_4_ at 30 °C (Fig. 4b). This is attributed to either increased expression and secretion or improved quality of the antibody, or both. The higher productivity of scFv in the strains (Nos. 6 and 7) is attributed to the productivity per cell rather than cell growth. Further work will be needed to determine whether increased expression requires genomic integration in general or at the *INU1* locus specifically. It is formally possible that episomal plasmids are not well retained during outgrowth, especially if protein expression creates stress for the host cell.

In our hands, YPX medium induced increased expression from the *INU1* promoter but not from the other tested promoters (Fig. 4a, b). As the *INU1* product inulinase metabolizes inuline to fructose (Rouwenhorst et al. 1988), *INU1* is downregulated in the presence of glucose, the preferred sugar (Jain et al. 2012). Moreover, *INU1* gene expression is known to be up-regulated when fructose replaces glucose as a sugar source (Schabort et al. 2016), and the *INU1* promoter has consensus binding sequences for MIG1, a known repressor of transcription in the presence of glucose (Bergkamp et al. 1993). As inulinase is the predominant protein secreted by *K. marxianus* (Rouwenhorst et al. 1990; Hu et al. 2012), deletion of the encoding locus is expected to permit re-direction of resources for expression and secretion of heterologous proteins. The present work showed that deletion of *INU1* had a positive but limited impact on scFv production in strain No. 8 (Km02-066). This limited effect may have reflected the use of the KmP_*MDH1*_ promoter and the Scα-MFss signal peptide. In *Kluyveromyces lactis*, the *Trichoderma reesei* CBH1 secretion signal was more efficient than that of the native α-mating factor for directing the secretion of a reporter, enhanced green fluorescent protein (EGFP) (Madhavan and Sukumaran 2014). Use of the endogenous INU1 signal peptide in *K. marxianus* may provide more efficient production by directing the heterologous protein into the secretion pathway typically used by inulinase.

In *K. marxianus*, lysine aminopeptidase activity is higher at 30 °C than at 20 °C (Ramírez-Zavala et al. 2004). It would be valuable to assess the in vivo role of various processing enzymes, for instance by suppressing the activity of endogenous proteases by using either protease inhibitors or genetic manipulations. In this study, however production of scFv increased as temperature elevated, suggesting that the proteases did not seriously affect scFv production in the conditions tested.

A positive effect of MgSO_4_ was observed (to some extent) in all strains and conditions, indicating that MgSO_4_ generally facilitates the production/secretion of intact scFv in *K marxianus* (Fig. 4a, b). Considering the concentration of MgSO_4_ in the defined media that typically ranges up to 10 or so, 200 mM of MgSO_4_ apparently exceeded the nutritional demands of the cell. The addition of divalent metal ions, including Mg^2+^, has been reported to enhance bacterial cell growth and enzyme production (Venkateswarulu et al. 2017; Shahbazmohammadi and Omidinia 2017). The effect of divalent metal ions may result from changes to membrane permeability (Venkateswarulu et al. 2017). In the present study, we observed drastic increases in antibody secretion in the presence of 200 mM MgSO_4_, a concentration that is ten times higher than that tested in bacteria. While fungal protein secretion pathways differ from those of bacteria, high concentrations of MgSO_4_ may also affect membrane organization in eukaryotes, facilitating protein secretion and/or stimulating expression of genes that contribute to enhanced protein production and secretion. The effect of MgSO_4_ significantly differed dependent on strain background, suggesting that it is implicated in the specific cellular processes. Most prominent effect was observed in the genomic integration strain in which scFv replaced *INU1* coding sequence and was expressed under *INU1* promoter with INU1 signal peptide, implying that MgSO_4_ has great impact on inulinase secretion pathway. However, MgSO_4_ is also likely to be involved in a wide range of fungal cellular and biochemical processes, the exact mechanism of this MgSO_4_-mediated enhancement of scFv production remains unclear. Nonetheless, our study demonstrated that there is potential for further enhancing fungal protein production by both genetic and physiological manipulations.

## Additional file

**Additional file 1: Table S1.** Primers used in this study. **Figure S1.** Amino acid sequence of scFv. **Figure S2.** Sequence of codon optimized scFv fragment. **Figure S3.** Sequence of E02-014 plasmid. **Figure S4.** Sequence of KmP*MDH1*. **Figure S5.** Sequence of KmP*ACO1*. **Figure S6.** Secreted scFv activity per cell amount.

## Abbreviations

TCA: tricarboxylic acid
scFv: single-chain antibody
CRISPR: Clustered Regularly Interspaced Short Palindromic Repeat
CRISPR–Cas9: Clustered Regularly Interspaced Short Palindromic Repeat (CRISPR)—associated protein
CHO: Chinese hamster ovary
IgGs: large immunoglobulins
HyHEL-10: anti-chicken (anti-hen) egg white lysozyme antibody
PVDF: polyvinylidene fluoride
NHEJ: non-homologous end-joining
EGFP: enhanced green fluorescent protein
