# Supplementary material for: Genetic and physiological basis for antibody production by Kluyveromyces marxianus
Source: AMB Express. 2018 Apr 12;8:56. doi: 10.1186/s13568-018-0588-1 (PMC5897269; doi:10.1186/s13568-018-0588-1)
Supplement: Supplementary file 1 — Additional file 1: Table S1. Primers used in this study. Figure S1. Amino acid sequence of scFv. Figure S2. Sequence of codon optimized scFv fragment. Figure S3. Sequence of E02-014 plasmid. Figure S4. Sequence of KmPMDH1. Figure S5. Sequence of KmPACO1. Figure S6. Secreted scFv activity per cell amount. [file 13568_2018_588_MOESM1_ESM.pdf]

AMB Express

Genetic and physiological basis for antibody production by *Kluyveromyces marxianus*

Yumiko Nambu-Nishida [1,2], Keiji Nishida [3], Tomohisa Hasunuma [3], Akihiko Kondo [2,3]*

[1] Technology Research Association of Highly Efficient Gene Design (TRAHED), 7-1-49 Minatojimaminamimachi, Chuo-ku, Kobe, Hyogo 650-0047, Japan

[2] Department of Chemical Science and Engineering, Graduate School of Engineering, Kobe University, 1-1 Rokkodai-cho, Nada-ku, Kobe, Hyogo 657-8501, Japan

[3] Graduate School of Science, Technology and Innovation, Kobe University, 1-1 Rokkodai-cho, Nada-ku, Kobe, Hyogo 657-8501, Japan

*Corresponding author

E-mail address: akondo@kobe-u.ac.jp (A. Kondo)

Tel.: +81-78-803-6196

Fax: +81-78-803-6196

Table S1. Primers used in this study

Primer name	Sequence (5'-3')	Purpose
Mdh1(Nhe1) (In-fusion)_Fw	GCGGCGCGCCGCTAGCGCATGCACCATGTTACCCG	amplification of KmP_{MDH1} region
Mdh1(Nhe1) (In-fusion)_Rv	CAGGTCCGGAGCTAGCTGTGTAGTAGTATATGTATGTACTTGG	
Aco1(Nhe1) (In-fusion)_Fw	GCGGCGCGCCGCTAGCGCTCCGATCCTCGGAGGC	amplification of KmP_{ACO1} region
Aco1(Nhe1) (In-fusion)_Rv	CAGGTCCGGAGCTAGCTATCAACAGATGATATGGTTTGTTG	
P_Km02-001 (INU(Km)_SPs_Fw)	AGCTAGCTCCGGACCTGcaggGATATGAAGTTAGCATACTCCCT	amplification of KmSUC2ss region
P_Km02-002 (INU(Km)_SPs_Rv)	TTGGATGTCGGATCCTCTCTTGTAATTGATCACTGAAGC	
P_Km02-003 (a-MF(Sc)_SPs_Fw)	AGCTAGCTCCGGACCTGcaggGATATGAGATTTCCTTCAATTTTTACTG	amplification of Scα-MFss region
P_Km02-005 (a-MF(Sc)kex2_SPs_Rv)	TTGGATGTCGGATCCTCTTTTATCCAAAGATACCCCTTC	
P_Km02_007 (single1(INU1)_2Rv)	CTAGCTCTAAAACATGGAAGCAAGAGGGAGTATGATTCGAACTGCGGACGTTG	amplification of target gRNA-3
P_Km02_008 (deletion(INU1)_3Fw)	CTAGCTCTAAAACGGTCACAGCGACATTTTATAGATTCGAACTGCGGACGTTG	amplification of target gRNA-1
P_Km02_009 (deletion(INU1)_4Fw)	CTAGCTCTAAAACCAGGTATCCGGTTGTAGTTGGATTCGAACTGCGGACGTTG	amplification of target gRNA-2
P_Km02_010 (Insert check)Fw	CTCGAGCGATCTTTCTTTCG	amplification of SUC2 gene target region
P_Km02_011 (Insert check)Rv	TAGGCAATCCCAAGGTAAGC	

* Red indicates each target sequence.

DIQLQESGPSLVKPSQTLSLTCSVTGDSITSDYWSWIRKFPGNRLEYMGYVSYSGSTYYNPSLKSRISITRDTSKNQ
YYLDLNSVTTEDTATYYCANWDGDYWGQGTLVTVSAGGGGSGGGGSGGGGSDIVLTQSPATLSVTPGNSVSLSCRAS
QSIGNNLHWYQQKSHESPRLLIKYASQSISGIPSRFSGSGSGTDFTLSINSVETEDFGMYFCQQSNSWPYTFGGGTK
LEYKHHHHHH*

Figure S1. Amino acid sequence of scFv
Orange: V_H, Green: V_L, Red: 6-His-tag

GACATCCAATTGCAAGAATCTGGTCCATCCTTGGTTAAGCCATCCCAAACCTTGTCTTTGACTTGTTCCGTCACCGG
TGACTCTATCACTTCCGATTACTGGTCTTGGATTAGAAAGTTCCCTGGTAACAGATTGGAATACATGGGTTACGTTT
CTTACTCCGGTTCTACCTACTACAACCCATCTTTGAAGTCCAGAATCTCTATTACCAGAGACACTTCCAAGAACCAA
TACTACTTGGACTTGAACTCTGTCACCACTGAAGATACCGCTACTTACTACTGTGCCAACTGGGACGGTGACTACTG
GGGTCAAGGTACCTTGGTTACTGTCTCCGCTGGTGGTGGTGGTTCTGGTGGTGGTGGTTCTGGTGGTGGTGGTAGTG
ACATCGTTTTGACTCAATCCCCAGCTACTTTGTCTGTTACCCCTGGTAACTCCGTCTCTTTGTCCTGTAGAGCCTCT
CAATCCATCGGTAACAACTTGCACTGGTACCAACAAAAGTCCCATGAATCTCCAAGATTGTTGATTAAGTACGCCTC
TCAATCCATCTCTGGTATTCCATCTAGATTCTCCGGTTCTGGTTCCGGTACCGACTTCACTTTGTCCATTAACTCTG
TTGAAACCGAAGATTTCGGTATGTACTTCTGTCAACAATCTAACTCCTGGCCATACACTTTCGGTGGTGGTACCAAG
TTGGAATACAAGCACCATCACCATCACCATTAA

Figure S2. Sequence of codon optimized scFv fragment

Orange: V_H, Green: V_L, Red: 6-His-tag

GTGAATTTACTTTAAATCTTGCATttaaataaattttctttttatagctttatgacttagtttcaattta
tatactattttaatgacattttcgattcattgattgaaagctttgtgtttttttcttgatgcgctattgca
ttgttcttgtcttttttcgccacatgtaatatctgtagtagatacctgatacattgtggatgctgagtgaa
attttagttaataatggaggcgctcttaataattttggggatattggctttttttttttaaagtttacaaa
tgaatttttttccgccaggatGCGGCCGCgaattcagggatgatcttgagaagttcttagagtcttacgag
ggaacagaagatttggaaccagccaaagctgctatagcggaagcagatattttgctaagtaaatgattaa
ataattaaatatgtggaaatacattaatctttttatatttttgcagttcgttgtcgctataatttatagt
catctcgttagttcaaacaagacttcttgaagtgaaaaccaactttcagtcttcaaactaaaaatgaaaa
tcagtggaagaaggtaaacgacttcatgttatatatgaattgaatagtaatggaaataaccaaaaacagc
tcaacagaaaacaaacaaaatacgttaagacctgaactcctagcagaaccataactgccaaatatttatt
atctgtggagatcttatattctaaaaccaaaaaaaatataacttaaaagttaaaaagaagatgttctaac
tgaggttcgaactcaggacctttgccgtgtgaaggcaacgtgatagccactacactattagaactacctt
atgggaaaaagaaaaatagagtacaactagaatggtaagatctgtgaccttttctaaacacttaattcca
tatagacagttcccacccaccataaggtcacaattataatgtctttagaagaccactgtcgttcatcatc
ttcctaagccctctctctaaagcggcatatttccgtaatttgttcttctttgcacaggcacgtgagatga
ctccgattattcccacatgcatatttagcCTCTCTAGGGGCTCGAGCTATAGCAAGTTCAAGGAAAGaaa
acTATTATGATCTGGTCACGTGTATAAAATTTATTAATTTTAAAACTATATAATTTATTATTTTTTTATT
TTAAAGTTTAAAGTAATTTTAATAGTATTCTATATTTTAAATAAACATACTTTAAAATTTTATTTAATAA
TTTATTATTTTTAAATACAATGTTTTTATTTAAAACAAAATTATAAGTTAAAAAGTTGTTCCGAAAGTAA
AATATATTTTATAGgacgtcGACATGGAGGCCcagaataccctccttgacagtcttgacgtgcgcagctc
aggggcatgatgtgactgtcgcccgtacatttagcccatacatccccatgtataatcatttgcatccata
cattttgatggccgcacggcgcgaagcaaaaattacggctcctcgctgcagacctgcgagcagggaaacg
ctcccctcacagacgcgttgaattgtccccacgccgcgcccctgtagagaaatataaaaggttaggattt
gccactgaggttcttctttcatatacttccttttaaaatcttgctaggatacagttctcacatcacatcc
gaacataaacaaccatgggtaaggaaaagactcacgtttcgaggccgcgattaaattccaacatggatgc
tgatttatatgggtataaatgggctcgcgataatgtcgggcaatcaggtgcgacaatctatcgattgtat
gggaagcccgatgcgccagagttgtttctgaaacatggcaaaggtagcgttgccaatgatgttacagatg
agatggtcagactaaactggctgacggaatttatgcctcttccgaccatcaagcattttatccgtactcc
tgatgatgcatggttactcaccactgcgatccccggcaaaacagcattccaggtattagaagaatatcct
gattcaggtgaaaatattgttgatgcgctggcagtgttcctgcgccggttgcattcgattcctgtttgta
attgtccttttaacagcgatcgcgtatttcgtctcgctcaggcgcaatcacgaatgaataacggtttggt
tgatgcgagtgattttgatgacgagcgtaatggctggcctgttgaacaagtctggaaagaaatgcataag
cttttgccattctcaccggattcagtcgtcactcatggtgatttctcacttgataaccttatttttgacg
aggggaaattaataggttgtattgatgttggacgagtcggaatcgcagaccgataccaggatcttgccat
cctatggaactgcctcggtgagttttctccttcattacagaaacggctttttcaaaaatatggtattgat
aatcctgatatgaataaattgcagtttcatttgatgctcgatgagtttttctaatcagtactgacaataa

aaagattcttgttttcaagaacttgtcatttgtatagtttttttatattgtagttgttctattttaatca
aatgttagcgtgatttatattttttttcgcctcgacatcatctgcccagatgcgaagttaagtgcgcaga
aagtaatatcatgcgtcaatcgtatgtGAATGCTGGTCGCTATACTGGAGCTCCAGCTTTTGTTCCCttt
agtgagggttaattgcgcgcttggcgtaatcatggtcatagctgtttcctgtgtgaaattgttatccgct
cacaattccacacaacataggagccggaagcataaagtgtaaagcctggggtgcctaatgagtgaggtaa
ctcacattaattgcgttgcgctcactgcccgctttccagtcgggaaacctgtcgtgccagctgcattaat
gaatcggccaacgcgcggggagaggcggtttgcgtattgggcgctcttccgcttcctcgctcactgactc
gctgcgctcggtcgttcggctgcggcgagcggtatcagctcactcaaaggcggtaatacggttatccaca
gaatcaggggataacgcaggaaagaacatgtgagcaaaaggccagcaaaaggccaggaaccgtaaaaagg
ccgcgttgctggcgtttttccataggctccgcccccctgacgagcatcacaaaaatcgacgctcaagtca
gaggtggcgaaacccgacaggactataaagataccaggcgtttccccctggaagctccctcgtgcgctct
cctgttccgaccctgccgcttaccggatacctgtccgcctttctcccttcgggaagcgtggcgctttctc
atagctcacgctgtaggtatctcagttcggtgtaggtcgttcgctccaagctgggctgtgtgcacgaacc
ccccgttcagcccgaccgctgcgccttatccggtaactatcgtcttgagtccaacccggtaagacacgac
ttatcgccactggcagcagccactggtaacaggattagcagagcgaggtatgtaggcggtgctacagagt
tcttgaagtggtggcctaactacggctacactagaaggacagtatttggtatctgcgctctgctgaagcc
agttaccttcggaaaaagagttggtagctcttgatccggcaaacaaaccaccgctggtagcggtggtttt
tttgtttgcaagcagcagattacgcgcagaaaaaaaggatctcaagaagatcctttgatcttttctacgg
ggtctgacgctcagtggaacgaaaactcacgttaagggattttggtcatgagattatcaaaaaggatctt
cacctagatccttttaaattaaaaatgaagttttaaatcaatctaaagtatatatgagtaaacttggtct
gacagttaccaatgcttaatcagtgaggcacctatctcagcgatctgtctatttcgttcatccatagttg
cctgactccccgtcgtgtagataactacgatacgggagggcttaccatctggccccagtgctgcaatgat
accgcgagacccacgctcaccggctccagatttatcagcaataaaccagccagccggaagggccgagcgc
agaagtggtcctgcaactttatccgcctccatccagtctattaattgttgccgggaagctagagtaagta
gttcgccagttaatagtttgcgcaacgttgttgccattgctacaggcatcgtggtgtcacgctcgtcgtt
tggtatggcttcattcagctccggttcccaacgatcaaggcgagttacatgatcccccatgttgtgcaaa
aaagcggttagctccttcggtcctccgatcgttgtcagaagtaagttggccgcagtgttatcactcatgg
ttatggcagcactgcataattctcttactgtcatgccatccgtaagatgcttttctgtgactggtgagta
ctcaaccaagtcattctgagaatagtgtatgcggcgaccgagttgctcttgcccggcgtcaatacgggat
aataccgcgccacatagcagaactttaaaagtgctcatcattggaaaacgttcttcggggcgaaaactct
caaggatcttaccgctgttgagatccagttcgatgtaacccactcgtgcacccaactgatcttcagcatc
ttttactttcaccagcgtttctgggtgagcaaaaacaggaaggcaaaatgccgcaaaaaagggaataagg
gcgacacggaaatgttgaatactcatactcttcctttttcaatattattgaagcatttatcagggttatt
gtctcatgagcggatacatatttgaatgtatttagaaaaataaacaaataggggttccGCGGCGCGCCGC
TAGCGCTCCGATCCTCGGAGGCAGGGTCACCCCAGCCACCTGTTACCCAACCGAAATAGGAATAAAAAAA
TCCCCAAAAGAACAAACAAACTTTTCCCCCTTTGCCGAACCTCGGAAAAGACCACTTTTCAATGACAACC

CTTACTCGGCCCCGGTTTCCGGGTACGCTTCCGAATACCCTGCACATGCCCCCTCTGCATGGATGTATGG
AGAGGCCCTTCCTGCTTGCCCCATTGCAAAAAAAATATCACAAACATACCCCTACACTGCAGCCGTTTCT
TAGTGCAGTCCAATCACGTCGCGTATTTGTTCGTGATCACATAATCACAACTACAGCTCTTTCTAAGCAT
TGAAACCATGTCCTCTTGCATACGCTCCTACGGCACTAACCGCGCTGTTCTCTGTCTGGCCCTTGCGTCC
CAACCATGCCATGCTGCTTCCTAGACACACACACGCACTCAGCTCACCAGAGAACAAACAAAACTCCATT
TATTCAGATCGCTCCCGTCACTTCAGAAGAGAAAATTTTTTTTCCCATTCTTCTACTTGTGCTTCTGCTG
CGCTGCCTTCGTGTTTCGAGTTGTTTTTAGGTTGGGCGTTAGCCAGTATAATGAAATTGAGTGATCAATC
GGCGGCGAATCGATCAAATTGATGACTTTTTTTTTTTTTTTTTTTTTGGGAGGTAAATTACGTTGAAAGC
TTGTGCTTATTGTAGTTGTATCTCTATACAAACGTAAATTTTAATATGTACAGTAGTAATTGTAGTAATA
ATAATAATAATAATAATAACACAATTAATTGTATATATAAGGGATTTCCGATTGGCTCCGTCTATATATT
TTTTTTGTTCTTTGGGCAAGTATAAAAAGTTCGAATAGTTGTTGCAGTTTTTTGGGAGTCCGTATTTTTT
AGTTTATTATTAGTTTATAGGCTATTAGTTTGTTATAATTGTATACAATTCGTTTTAATCGAACAAGAAC
AACAAACCATATCATCTGTTGATAGCTAGCTCCGGACCTGcaggGATATGAAGTTAGCATACTCCCTCTT
GCTTCCATTGGCAGGAGTCAGTGCTTCAGTGATCAATTACAAGAGAGGATCCGACATCCAATTGCAAGAA
TCTGGTCCATCCTTGGTTAAGCCATCCCAAACCTTGTCTTTGACTTGTTCCGTCACCGGTGACTCTATCA
CTTCCGATTACTGGTCTTGGATTAGAAAGTTCCCTGGTAACAGATTGGAATACATGGGTTACGTTTCTTA
CTCCGGTTCTACCTACTACAACCCATCTTTGAAGTCCAGAATCTCTATTACCAGAGACACTTCCAAGAAC
CAATACTACTTGGACTTGAACTCTGTCACCACTGAAGATACCGCTACTTACTACTGTGCCAACTGGGACG
GTGACTACTGGGGTCAAGGTACCTTGGTTACTGTCTCCGCTGGTGGTGGTGGTTCTGGTGGTGGTGGTTC
TGGTGGTGGTGGTAGTGACATCGTTTTGACTCAATCCCCAGCTACTTTGTCTGTTACCCCTGGTAACTCC
GTCTCTTTGTCCTGTAGAGCCTCTCAATCCATCGGTAACAACTTGCACTGGTACCAACAAAAGTCCCATG
AATCTCCAAGATTGTTGATTAAGTACGCCTCTCAATCCATCTCTGGTATTCCATCTAGATTCTCCGGTTC
TGGTTCCGGTACCGACTTCACTTTGTCCATTAACTCTGTTGAAACCGAAGATTTCGGTATGTACTTCTGT
CAACAATCTAACTCCTGGCCATACACTTTCGGTGGTGGTACCAAGTTGGAATACAAGCACCATCACCATC
ACCATTAACCCGGGactagt

Figure S3. Sequence of E02-014 plasmid

Gray: ScT_{TDH3}, Blue: KmARS7, Green: KmCEN-D, Orange: *kanMX*, Light green: KmP_{ACO1}, Red: KmINU1ss, Pink: scFv

GCATGCACCATGTTACCCGGGGGGCATCCATGTCTCTCCCTTCCTCTTCCTCTTTTTTTACTAATTTCCC
TGTTGTCTTCCGTTTTTTTCTTTTTTTCCCCTATCGCCAATGCATGACCAAAGTCCTTTTTTTTTTTCCC
CATGACACAGCCCGTTGGAACACGCTGAAAACACGCTGAAGAAAAACAAGGGAAACACTGGAACAGTGAA
ACACCCGCACGAAAATGTTCGGAGTCGGCTCCGCGTGGTCCCAATTAATAAACAACGTAGCAGCTAGCCA
GCTTTAAACTGACTTAGTCCTACGGTTTTGCTGCGTGGGGGAACCGAGGGAGGGAATCCTCTGGAAGCCG
GACCTTGCCCTTTTGACTAGGGTACTACTCTGGGCAGGGTACTTACTAAACTGGGCCCAGACAGACAGAG
GCCCAGACAGAGGCCCCAAAGAGAAGCCGCCAGGTTCCCTGCCTAGGCCTTCCTCTGGTTGCCCCCCAGA
GTACCCTCCGGAACGAAACGAAACGAAAACGCACATCGTGATGCGCGCTATTATAATTGCGTCTTGCGAA
TTCCATACGCCATGCGCTTAGTTAGCACATGAAATCCTGCCAATATTGACAATCCAACTTCCCAATTCCA
TTCCCAATTCCATTCCTACCTAATTCCAGTTCCCATTCATGACCACCGCCGACTCGCCCACAGTTGGCCC
ACAATTGGCCCATATAATATCAGGATATCAGAAAAATAGTTACATATATACAAACAAATTGAAAAATATA
TATATTATATATTATGGATATTAATATTATATATATATATATGTATATATATCATGACATAAAGCTTTGA
TTGATAATAATACCTCTACTTCTTGGTATGATAATGTGATCTCTTGCTATCTTATTCCTTTATCTAACAC
CCCCCCCAAGTACATACATATACTACTACACA

Figure S4.   Sequence of KmP_{MDH1}

GCTCCGATCCTCGGAGGCAGGGTCACCCCAGCCACCTGTTACCCAACCGAAATAGGAATAAAAAAATCCC
CAAAAGA ACAAACAAACTTTTCCCCCTTTGCCGAACCTCGGAAAAGACCACTTTTCAATGACAACCCTT
ACTCGGCCCCGGTTTCCGGGTACGCTTCCGAATACCCTGCACATGCCCCCTCTGCATGGATGTATGGAGA
GGCCCTTCCTGCTTGCCCCATTGCAAAAAAAATATCACAAACATACCCCTACACTGCAGCCGTTTCTTAG
TGCAGTCCAATCACGTCGCGTATTTGTTCGTGATCACATAATCACAACTACAGCTCTTTCTAAGCATTGA
AACCATGTCCTCTTGCATACGCTCCTACGGCACTAACCGCGCTGTTCTCTGTCTGGCCCTTGCGTCCCAA
CCATGCCATGCTGCTTCCTAGACACACACACGCACTCAGCTCACCAGAGAACAAACAAAACTCCATTTAT
TCAGATCGCTCCCGTCACTTCAGAAGAGAAAATTTTTTTTCCCATTCTTCTACTTGTGCTTCTGCTGCGC
TGCCTTCGTGTTTCGAGTTGTTTTTAGGTTGGGCGTTAGCCAGTATAATGAAATTGAGTGATCAATCGGC
GGCGAATCGATCAAATTGATGACTTTTTTTTTTTTTTTTTTTTTGGGAGGTAAATTACGTTGAAAGCTTG
TGCTTATTGTAGTTGTATCTCTATACAAACGTAAATTTTAATATGTACAGTAGTAATTGTAGTAATAATA
ATAATAATAATAATAACACAATTAATTGTATATATAAGGGATTTCCGATTGGCTCCGTCTATATATTTTT
TTTGTTCTTTGGGCAAGTATAAAAAGTTCGAATAGTTGTTGCAGTTTTTTGGGAGTCCGTATTTTTTAGT
TTATTATTAGTTTATAGGCTATTAGTTTGTTATAATTGTATACAATTCGTTTTAATCGAACAAGAACAAC
AAACCATATCATCTGTTGATA

Figure S5. Sequence of KmP_{ACO1}

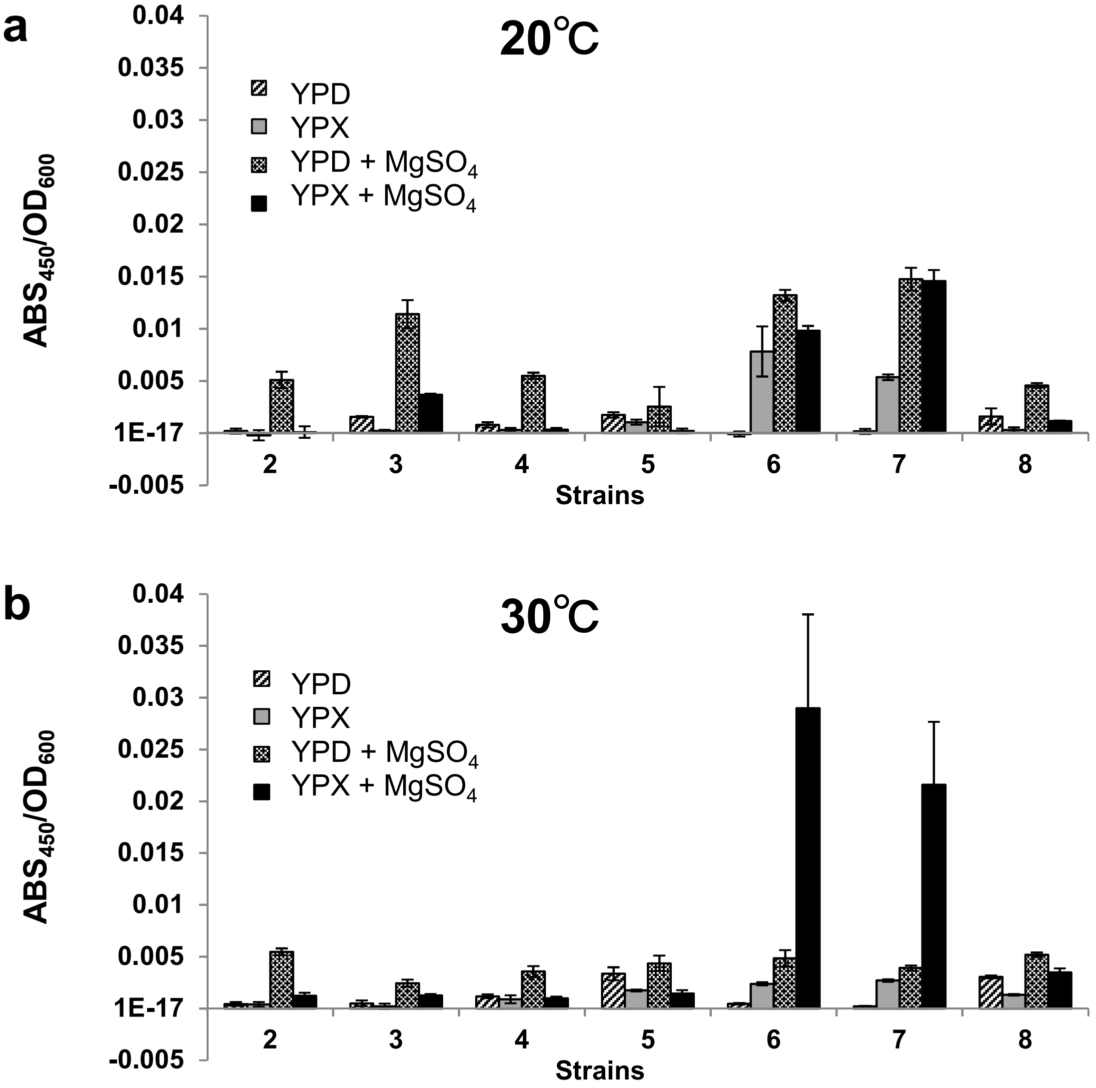

Figure S6. Secreted scFv activity per cell amount

Strains were cultured in YPD or YPX in the absence or presence of $MgSO_4$ at 20°C (a) or 30°C (b). The value is calculated as scFv activity (ABS_{450}) per cell amount (OD_{600}). Values are presented as mean ±SEM from three independent experiments.
